# Effects of Temperature and Salinity on Seed Germination of Three Common Grass Species

**DOI:** 10.3389/fpls.2021.731433

**Published:** 2021-12-10

**Authors:** Yongjie Liu, Shuang Zhang, Hans J. De Boeck, Fujiang Hou

**Affiliations:** ^1^State Key Laboratory of Grassland Agro-Ecosystems, Key Laboratory of Grassland Livestock Industry Innovation, Ministry of Agriculture and Rural Affairs, College of Pastoral Agriculture Science and Technology, Lanzhou University, Lanzhou, China; ^2^Plants and Ecosystems (PLECO), Department of Biology, University of Antwerp, Wilrijk, Belgium

**Keywords:** germination percentage, germination rate, grass species, salinity, temperature

## Abstract

Temperature and salinity significantly affect seed germination, but the joint effects of temperature and salinity on seed germination are still unclear. To explore such effects, a controlled experiment was conducted, where three temperature levels (i.e., 15, 20, and 25°C) and five salinity levels (i.e., 0, 25, 50, 100, and 200 mmol/L) were crossed, resulting in 15 treatments (i.e., 3 temperature levels × 5 salinity levels). Three typical grass species (*Festuca arundinacea*, *Bromus inermis*, and *Elymus breviaristatus*) were used, and 25 seeds of each species were sown in petri dishes under these treatments. Germination percentages and germination rates were calculated on the basis of the daily recorded germinated seed numbers of each species. Results showed that temperature and salinity significantly affected seed germination percentage and germination rate, which differed among species. Specifically, *F. arundinacea* had the highest germination percentage, followed by *E. breviaristatus* and *B. inermis*, with a similar pattern also found regarding the accumulated germination rate and daily germination rate. Generally, *F. arundinacea* was not sensitive to temperature within the range of 15–25°C, while the intermediate temperature level improved the germination percentage of *B. inermis*, and the highest temperature level benefited the germination percentage of *E. breviaristatus*. Moreover, *F. arundinacea* was also not sensitive to salinity within the range of 0–200 mmol/L, whereas high salinity levels significantly decreased the germination percentage of *B. inermis* and *E. breviaristatus*. Thus, temperature and salinity can jointly affect seed germination, but these differ among plant species. These results can improve our understanding of seed germination in saline soils in the face of climate change.

## Introduction

Seed germination is a fundamental stage in the life cycle of a plant (Bewley, 1997; Nimbalkar et al., 2020). Seed germination is significantly affected by both physical and biological factors such as temperature and species identity (Larsen et al., 2004; Bewley et al., 2013; Zhang et al., 2020). Soil salinization is one of the major drivers of soil degradation (Zhang et al., 2015a; Gorji et al., 2017), and it can significantly affect seed germination and the following stages such as seedling establishment (Khan and Gulzar, 2003; Qu et al., 2008). Over 900 Mha land is impacted by salinity in the whole world (Rengasamy, 2006; Shiade and Boelt, 2020). Climate change such as extreme warming is expected to be more frequent in the future (Khan and Qaiser, 2006; Blackport and Screen, 2020; Bai et al., 2021). Such change could significantly affect seed germination (Walck et al., 2011; Mondoni et al., 2012). Soil salinization could become more serious in the face of climate change because global warming generally increases evaporation, which can promote soil salinization (Utset and Borroto, 2001). Therefore, salinity and temperature would jointly affect seed germination, especially in the arid and semi-arid areas of northeastern China, where the soil salinization area covers over 70% of the total land area (Wang et al., 2011). Moreover, several species are facing population reductions due to human disturbances and climate change (Richmond et al., 2007; Ureta et al., 2012; Gu et al., 2018). Thus, exploring seed germination under the ongoing soil salinization and global warming is important in assessing the stability of plant community.

Theoretically, the seed germination of each species has an optimal temperature, under which seeds could germinate better than under other temperatures. Previous studies found that salinity decreased seed germination of some species compared with non-saline conditions (Khan and Gulzar, 2003; Qu et al., 2008). However, the impact of salinity on seed germination might be modified by temperature, as Gorai and Neffati (2007) found that negative effects of salinity on seed germination were less severe at the optimum temperature, as the additional environmental stress at low or high temperatures would thus be alleviated (Al-Khateeb, 2006). Yet, Khan and Ungar (2001) found that the effect of salinity was stronger at lower temperatures, while Delesalle and Blum (1994) revealed that such effect was stronger at higher temperatures. Finally, Khan and Ungar (1998) showed that the effect of salinity was not affected by temperature in their experiment. Thus, the joint effects of salinity and temperature on seed germination are still unclear (Fernandez et al., 2015; Lin et al., 2018).

In response to local salinity and suboptimal temperatures, plant species developed different strategies, including adjusting germination percentage or germination rate through modifying seed dormancy and/or seed viability (Ungar, 1995; Khan et al., 2001; Khan and Ungar, 2001; Shahba et al., 2008; Guan et al., 2009). Such responses can further alter seedling establishment and seedling growth (Gu et al., 2018; Del Vecchio et al., 2021). Exploring the effects of salinity and temperature on seed germination may shed light on understanding the mechanisms of species coexistence. However, studying such effects under natural conditions is difficult since (1) soil conditions such as temperature and salinity vary spatially and temporally (Hermans et al., 2016), which makes it difficult to keep a constant level of temperature or salinity. (2) Other environmental variables such as radiation and soil moisture hamper separating the roles of temperature and salinity from these factors (Khan and Ungar, 1997; De Boeck et al., 2015; Borja et al., 2016; Bhatt et al., 2020). (3) Some particular species in a community such as halophytes and xerophytes may skew the results, where halophytes can modify their strategies (e.g., reduce seed germination percentage or delay the start of germination under the high level of salinity) to adapt to different salinity levels (Gulzar and Khan, 2001; Khan and Gul, 2006; El-Keblawy et al., 2020), and xerophytes can grow well under conditions with a large variation of temperature (Zhang et al., 2015b).

**FIGURE 1:**
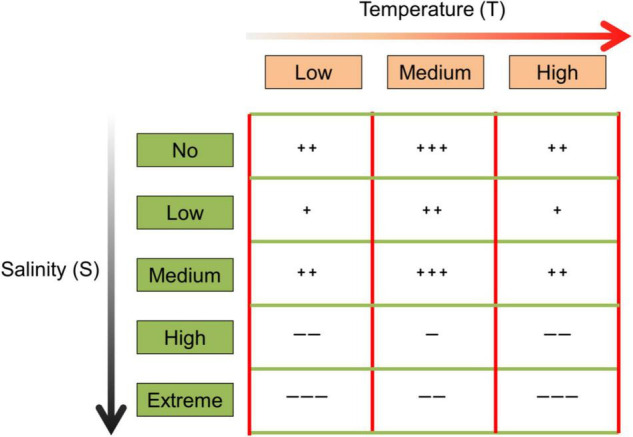
The expected effects of temperature (three levels: low, medium, and high) and salinity (five levels: no, low, medium, high, and extreme) on seed germination, where “+” and “–” refer to the positive and negative effect, respectively. More “+” or “–” indicates a stronger effect.

To explore the joint effects of temperature and salinity on seed germination of grass species with less confounding factors (Figure 1), a controlled experiment was thus conducted. Three typical grass species (*Festuca arundinacea*, *Bromus inermis*, and *Elymus breviaristatus*) widely used as forage species (Lu et al., 2008) that can be potentially grown in saline soils were exposed to three levels of temperature and five levels of salinity. Specifically, (1) we expect seed germination in general to be the highest at the intermediate level of temperature (20°C), which is thought to be closest to the optimal temperature for seed germination for such grasses (Romo and Eddleman, 1995; Lu et al., 2008; Zhang et al., 2013). (2) We assume that seed germination would consistently decrease with increasing salinity (Wu et al., 2015; Zhang and Dai, 2019). (3) We anticipate that the intermediate (and supposed optimum) temperature level would alleviate the negative effects of salinity on seed germination (Gorai and Neffati, 2007).

## Materials and Methods

### Experimental Design

To explore the effects of temperature and salinity on seed germination, an experiment was conducted at the Yuzhong Campus of Lanzhou University, China (104°09′44″N, 35°56′55″E) from 6 April to 25 April 2021. Three levels of temperature (i.e., 15, 20, and 25°C) and five levels of salinity (i.e., NaCl concentration 0, 25, 50, 100, and 200 mmol/L) were created to simulate the future climatic conditions. Note that these temperature and salinity levels were set in line with previous studies (Lu et al., 2008 and Zhang et al., 2013 for temperature levels; Yang et al., 2009 and Li et al., 2019 for salinity levels). Three target grass species (*F. arundinacea*, *B. inermis*, and *E. breviaristatus*) were exposed to these 15 treatments. A recent study reported that different varieties of a species responded differently to salinity stress (Shiade and Boelt, 2020). However, this study aimed to explore the responses of seed germination of different species to the joint effects of temperature and salinity, not of varieties of specific species. Seeds of the three species used in our experiment were bought from a commercial company (Best, Beijing, China). Further information can be found in Table 1. Twenty-five seeds of each species were applied in each treatment. All seeds were evenly sown in petri dishes with two sheets of filter paper (diameter 7 cm). The filter paper was saturated with saline solutions (around 5 mL) and kept stable during the experiment.

**TABLE 1:** Information of the seeds applied in this experiment.

Species	Variety name	Standard germination percentage (%)	Seed color	1,000 grain weight (g)	Length (mm)	Width (mm)	Thickness (mm)
*Festuca arundinacea*	Niuniu	> 85	Dark gray	2.6 ± 0.1	7.0 ± 0.8	1.6 ± 0.1	0.9 ± 0.1
*Bromus inermis*	Normal	> 85	Brown	4.1 ± 0.1	9.5 ± 0.6	1.8 ± 0.1	0.7 ± 0.1
*Elymus breviaristatus*	Normal	> 80	Light gray	5.6 ± 0.1	11.7 ± 1.5	1.7 ± 0.1	1.7 ± 0.1

*“Normal” in the variety name reflects that there is no specific variety for this species.*

Three incubators (LRH-250-G, Illuminating Incubator) were used, and each of them was set at one of the three applied temperature levels. Petri dishes with the five salinity levels were randomly stored in each of these chambers. These petri dishes were covered with lids at the beginning of the experiment, and they were removed after the germination of the seeds since lids impeded the growth of these seedlings. Five replicates were used per treatment, resulting in 225 petri dishes (i.e., 3 species × 3 temperature levels × 5 salinity levels × 5 replications) in total. Note that the seed germination test was conducted according to the rules of the International Seed Testing Associations (ISTA, 2018), and the germinated seeds in each petri dish were daily recorded. Seeds were treated as germinated when the radicle was more than 2 mm long (Shiade and Boelt, 2020). This experiment was ended when there was no additional germination for 3 days.

### Data Analysis and Statistics

Germination percentage (GP) was calculated by dividing the germinated seed number by the total seed number in each petri dish along the experimental period. Accumulated germination rate (AGR) and daily germination rate (DGR) in each petri dish were calculated by the following two equations:

AGR = (∑*GP*_*i*_)/*i*, where *i* is the day after seed set in these chambers;

DGR = the newly germinated seed number per day/25 in each petri-dish.

To explore the seed germination during the experiment, four separate analyses were conducted. First, repeated-measures ANOVA was used to explore the differences of GP, AGR, and DGR among the target species. Second, repeated-measures ANOVAs were applied to investigate the effects of temperature, species, and their interactions on the GP. Third, repeated-measures ANOVAs were employed to test the effects of salinity, species, and their interactions on the GP. A significant effect of species was found in the second and third analyses. Thus, separate repeated-measures ANOVAs analyses were conducted for each species, where temperature (or salinity), time, and their interaction were treated as variables. Fourth, MANOVA was performed to examine the impacts of temperature, salinity, species and their interactions on the GP, AGR at the last day of the experiment, and the average DGR during the experiment. Note that time (i.e., the germination date) was treated as an extra factor in these analyses except the last one.

Curve estimations were conducted to explore the relationships between salinity and GP separated by temperature, where linear, quadratic, power, and exponential curves were tested. A better model was identified with a lower Akaike Information Criterion (AIC) and a significant *P*-value. All statistics were performed with SPSS 23.0 (IBM Corp, 2015).

## Results

**FIGURE 2:**
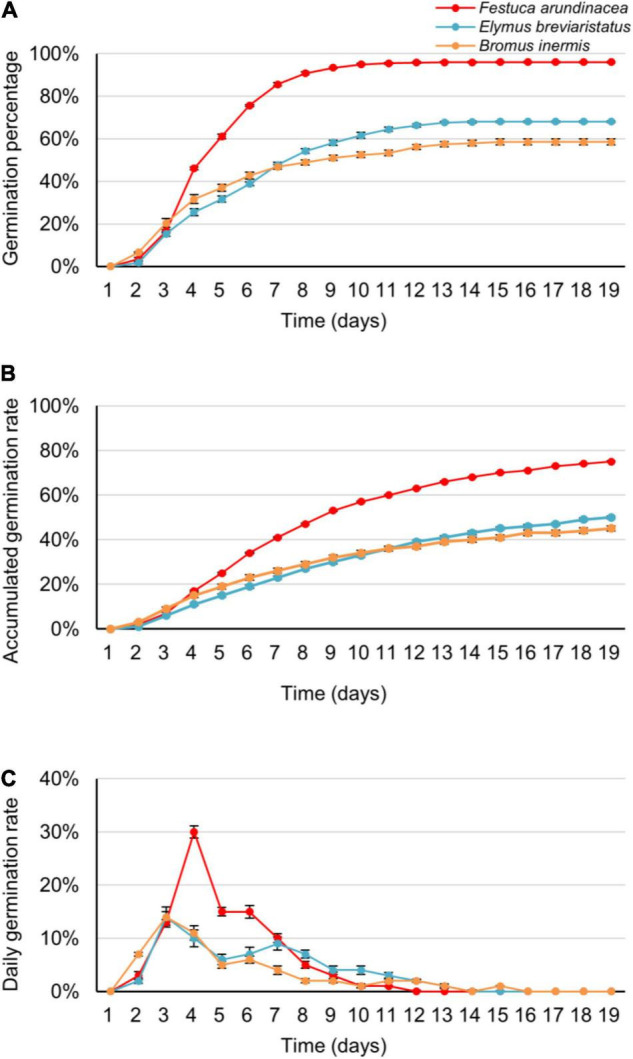
The germination percentage **(A)**, accumulated germination rate **(B)**, and daily germination rate **(C)** of the three target grass species (*Festuca arundinacea*, *Bromus inermis*, and *Elymus breviaristatus*, labeled as red, orange, and blue color, respectively) along time (i.e., the germination date). Note that these figures are derived from the average data of the three temperature levels and five salinity levels.

In the first analysis, GP, AGR, and DGR varied within species, germination date, and species × germination date interaction (Table 2 and Figure 2). On average, the GP of *F. arundinacea* was higher than that of *E. breviaristatus* and *B. inermis*, and the GP of *E. breviaristatus* was in turn higher than that of *B. inermis* (Figure 2A). Such a pattern was also found for AGR (Figure 2B) and DGR (Figure 2C). *B. inermis* germinated faster at the beginning of the experiment, while its germination decreased faster than the other two species during the experiment (Figure 2C). The interaction effect between species and germination date was likely caused by the convergence of the seed germination (Figure 2).

**TABLE 2:** Effects of species, time, and their interaction in repeated-measures ANOVA of germination percentage (GP), accumulated germination rate (AGR), and daily germination rate (DGR).

	GP			AGR			DGR		
	df	*F*	*P*	df	*F*	*P*	df	*F*	*P*
Species	2,144	345.4	** < 0.001**	2,144	192.7	** < 0.001**	2,144	333.7	** < 0.001**
Time	18,144	5304.4	** < 0.001**	18,144	14991.4	** < 0.001**	18,144	163.7	** < 0.001**
Species × Time	36,144	164.5	** < 0.001**	36,144	267.9	** < 0.001**	36,144	20.4	** < 0.001**

*F-values, P-values, and degrees of freedom (df_between–groups_, df_within–groups_) are given with significant results (P < 0.05) in bold.*

**FIGURE 3:**
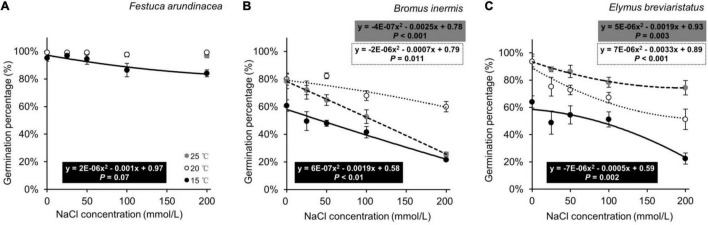
Seed germination percentages of *Festuca arundinacea* **(A)**, *Bromus inermis* **(B)**, and *Elymus breviaristatus* **(C)** under different temperatures as a function of salinity levels. Note that all these significant equations are non-linear, so *P*-values are given.

In the second analysis, on investigating the effects of species, temperature, and their interaction on GP, the three target species demonstrated different responses (Table 3 and Figure 3). The GP of *F. arundinacea* was not sensitive to the relatively high levels of temperature (Figure 3A). The GP of *B. inermis* was highest at the intermediate temperature level (Figure 3B), and the GP of *E. breviaristatus* was highest at the highest temperature level in this study (Figure 3C).

**TABLE 3:** Effects of species, temperature, time, and their interactions in repeated-measures ANOVA of germination percentage, which was separated by species since it was a significant factor.

Source	Germination percentage		
	df	*F*	*P*
Species	2,384	281.5	** < 0.001**
Temperature	2,384	661.7	** < 0.001**
Time	18,384	4309.1	** < 0.001**
Species × Temperature	4,384	10.5	**0.001**
Species × Time	36,384	122.8	** < 0.001**
Temperature × Time	36,384	155.8	** < 0.001**
Species × Temperature × Time	72,384	24.9	** < 0.001**
***Festuca arundinacea***			
Temperature	2,144	370.3	**< 0.001**
Time	18,144	4906.3	**< 0.001**
Temperature × Time	36,144	86.3	**< 0.001**
***Bromus inermis***			
Temperature	2,144	122.3	** < 0.001**
Time	18,144	650.3	** < 0.001**
Temperature × Time	36,144	43.3	** < 0.001**
***Elymus breviaristatus***			
Temperature	2,144	75.5	** < 0.001**
Time	18,144	2332.6	** < 0.001**
Temperature × Time	36,144	28.0	** < 0.001**

*F-values, P-values, and degree of freedom (df_between–groups_, df_within–groups_) are given with significant results (P < 0.05) in bold.*

**FIGURE 4:**
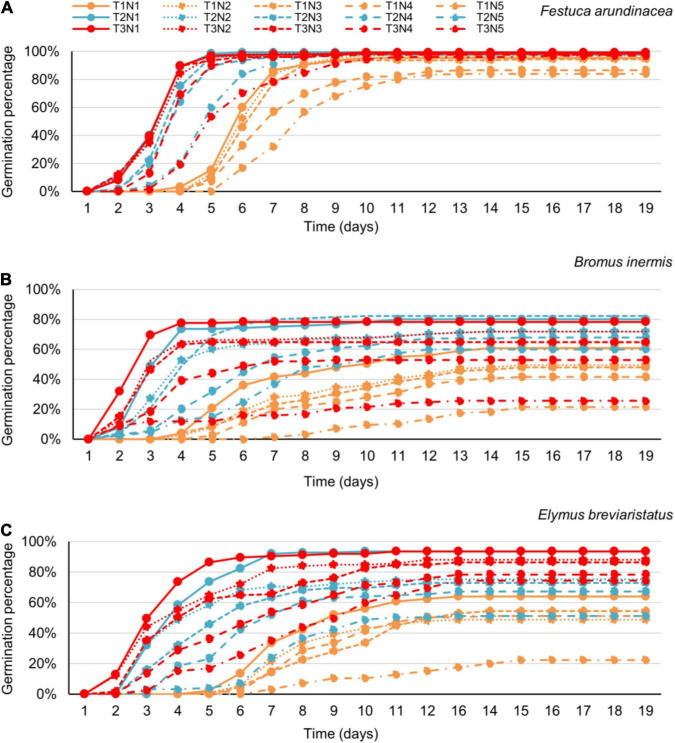
The joint effect of temperature and salinity on seed germination of *Festuca arundinacea* **(A)**, *Bromus inermis* **(B)**, and *Elymus breviaristatus* **(C)** as a function of time (i.e., the germination date). Note that T1–T3 refer to the three temperature levels, that is, 15, 20, and 25°C, respectively, while N1–N5 reflect the five salinity levels, that is, 0, 25, 50, 100, and 200 mmol/L, respectively.

In the third analysis, on testing the effects of species, salinity, and their interaction on GP, the three target species likewise showed different patterns (Table 4 and Figure 3). The GP of *F. arundinacea* was not sensitive to relatively low levels of salinity. However, the other two species showed a different pattern, where the higher salinity levels decreased the GP of *B. inermis*, while the intermediate level of salinity increased. The GP of *E. breviaristatus* consistently decreased with increasing salinity levels. Moreover, the intermediate temperature level (i.e., 20°C) × lowest salinity level (i.e., 0 mmol/L) generated the highest GP for *F. arundinacea*, while the highest temperature level (i.e., 25°C) × lowest salinity level (i.e., 0 mmol/L) generated the highest GP for *B. inermis* and *E. breviaristatus* (Figure 4).

**TABLE 4:** Effects of species, salinity, time, and their interactions in repeated-measures ANOVA of germination percentage, which was separated by species since it was a significant factor.

Source	Germination percentage		
	Df	*F*	*P*
Species	2,720	380.4	** < 0.001**
Salinity	4,720	132.3	** < 0.001**
Time	18,720	4258.7	** < 0.001**
Species × Salinity	8,720	7.1	**0.001**
Species × Time	36,720	167.0	** < 0.001**
Salinity × Time	72,720	27.0	** < 0.001**
Species × Salinity × Time	144,720	5.7	** < 0.001**
***Festuca arundinacea***			
Temperature	4,288	24.0	** < 0.001**
Time	18,288	4829.5	** < 0.001**
Temperature × Time	72,288	13.8	** < 0.001**
***Bromus inermis***			
Temperature	4,288	57.0	** < 0.001**
Time	18,288	667.2	** < 0.001**
Temperature × Time	72,288	16.7	** < 0.001**
***Elymus breviaristatus***			
Temperature	4,288	34.4	** < 0.001**
Time	18,288	1266.5	** < 0.001**
Temperature × Time	72,288	9.4	** < 0.001**

*F-values, P-values, and degree of freedom (df_between–groups_, df_within–groups_) are given with significant results (P < 0.05) in bold.*

Finally, exploring the effects at the last day of the experiment, species, temperature, salinity, species × temperature, species salinity, and species × temperature × salinity significantly affected GP, AGR, and DGR (Table 5 and Figure 4), while there were no significant temperature × salinity effects at this measurement data.

**TABLE 5:** Effects of temperature, salinity, species, and their interactions in MANOVA of germination percentages (GP), accumulated germination rate (AGR), and daily germination rate (DGR).

	GP			AGR			DGR		
	df	*F*	*P*	df	*F*	*P*	df	*F*	*P*
Species	2,180	356.1	** < 0.001**	2,180	268.2	** < 0.001**	2,180	193.0	** < 0.001**
Temperature	2,180	102.3	** < 0.001**	2,180	268.2	** < 0.001**	2,180	193.0	** < 0.001**
Salinity	4,180	42.7	** < 0.001**	4,180	79.3	** < 0.001**	4,180	60.7	** < 0.001**
Species × Temperature	4,180	13.0	** < 0.001**	4,180	5.9	** < 0.001**	4,180	10.3	** < 0.001**
Species × Salinity	8,180	5.6	** < 0.001**	8,180	2.9	**0.004**	8,180	3.6	**0.001**
Temperature × Salinity	8,180	1.3	0.255	8,180	1.8	0.081	8,180	0.8	0.623
Species × Temperature × Salinity	16,180	1.6	0.080	16,180	1.1	0.335	16,180	2.4	**0.003**

*F-values, P-values, and degrees of freedom (df_between–groups_, df_within–groups_) are given, with significant results (P < 0.05) in bold.*

## Discussion

The first hypothesis stated that seed germination would be the highest at the intermediate level of temperature. This was partly supported as such a pattern was found in one of the target plant species (i.e., *B. inermis*, Figure 3B), where lower germination was found at lower temperatures. This is partly consistent with the finding of Ao et al. (2014), where seed germination of *B. inermis* was low at lower temperatures. Note that such a pattern was not found in the other two target species. For *F. arundinacea*, temperature levels in this study may have all been in the optimal temperature range of this species (Lu et al., 2008), while for *E. breviaristatus*, the optimal temperature of seed germination might have been higher than the temperature levels we set (Figure 3C).

Our second hypothesis aimed to test whether seed germination would be reduced at higher levels of salinity. This was supported as seed germination of the three target species was generally lower at higher salinity levels, even though they responded inconsistently to the salinity gradient (Figure 3). Such results are in line with previous studies on the target species *F. arundinacea* (Shiade and Boelt, 2020), *B. inermis* (Yang et al., 2009). and *E. breviaristatus* (Li et al., 2019), and on other species such as *Helianthus annuus* (Wu et al., 2015), *Oryza sativa* (Xu et al., 2011), and *Zea mays* (Khodarahmpour et al., 2012). Such results could be related to the effects of ion toxicity on seed germination (Panuccio et al., 2014). The different responses of plants to salinity are likely caused by the genetic traits of these species (Vu et al., 2015; Chamorro et al., 2017) and their growing conditions (Mira et al., 2017).

The last hypothesis focused on the joint effects of salinity and temperature on seed germination, and we expected that the negative effect of salinity on seed germination would be alleviated at the intermediate level of temperature. This was supported by our findings in one of the three target species (*B. inermis*, Figure 3B), where the germination percentage of *B. inermis* at the intermediate temperature level was higher than at the other two temperature levels, and the germination percentage decreased more slowly with increasing salinity compared with the other two temperature levels. This is in line with the finding of Gorai and Neffati (2007), where the negative effect of salinity on seed germination was alleviated at the optimum temperature. However, the other two species did not show such a pattern.

Results of this study should be interpreted and extrapolated with caution because of the following two reasons. One is that NaCl solutions in this study might evaporate at different rates when they were set under different temperatures during the experiment (Sayer et al., 2017), and this may affect the ultimate salinity level and thus the ensuing results. The other is that each level of temperature was kept constant during the experiment in this study, while previous studies found that variation of temperature can benefit seed germination (Liu et al., 2013, 2017a; Spindelböck et al., 2013; Burghardt et al., 2016). Moreover, soil resources such as soil temperature and salinity vary a lot even at a short distance in natural conditions (Maestre et al., 2003; Lundholm, 2010). Thus future studies on seed germination should consider the heterogeneous distributions of these factors, potentially in combination with other aspects of soil heterogeneity (e.g., Liu et al., 2017b,c, 2019; Liu and Hou, 2021).

## Data Availability Statement

The original contributions presented in the study are included in the article/supplementary material, further inquiries can be directed to the corresponding author.

## Author Contributions

YL designed the study, conducted the analyses, and wrote the first draft of the manuscript. SZ collected the data. All authors contributed significantly to the manuscript.

## Conflict of Interest

The authors declare that the research was conducted in the absence of any commercial or financial relationships that could be construed as a potential conflict of interest.

## Publisher’s Note

All claims expressed in this article are solely those of the authors and do not necessarily represent those of their affiliated organizations, or those of the publisher, the editors and the reviewers. Any product that may be evaluated in this article, or claim that may be made by its manufacturer, is not guaranteed or endorsed by the publisher.
